# Supplementary material for: Chromothripsis during telomere crisis is independent of NHEJ, and consistent with a replicative origin
Source: Genome Res. 2019 May;29(5):737–49. doi: 10.1101/gr.240705.118 (PMC6499312; doi:10.1101/gr.240705.118)
Supplement: Supplemental Material [file supp_gr.240705.118_Supplemental_file_1.zip › contigs/annotated_contigs/DB51/contig.2.DB51_length_287_mean_cov_3.65853658537.docx]

**DB51_length_287_mean_cov_3.65853658537**

```
CCTCACCTGCACTTTGTGCTCTAGCTTAGTACAACTCTTTGGCAGTTCTTAGAATCAGCCATGTTGGTTTTCATCCTTTTGTCTCCTCA
 >chr6:79766016-79766107 - E=2e-43 p=3e-02
CA|GTGTTT|CTTTGGAAGTAAATGCTGCTATGCCAAGTACACACTCACATCTCTCTTCCACAATAAAAGAATCACAAGCTAGTAATAA
         >chr6:79765204-79765394 + E=3e-103
CAACAGATCAGTGGGATCTTTTGTCTTTGCTTTTGAAAACAGTATTAAAGGAGGTTCTAGAGCACTGGAAGGCAGGTGAACCACTTTGG

GTCTCTTGCTGAGACTGAGTTC
```
